# *BMC Geriatrics* reviewer acknowledgement 2015

**DOI:** 10.1186/s12877-016-0219-0

**Published:** 2016-02-09

**Authors:** Giulia Mangiameli

**Affiliations:** BioMed Central, Floor 6, 236 Gray’s Inn Road, London, WC1X 8HB UK

## Abstract

The editors of *BMC Geriatrics* would like to thank all our reviewers who have contributed to the journal in Volume 15 (2015).

**Melanie Abas**

UK

**Edimansyah Abdin**

Singapore

**Wilco Achterberg**

Netherlands

**Odessa Addison**

USA

**Abraham Adunsky**

Israel

**Masami Akai**

Japan

**Sultan H. Alamri**

Canada

**Steven Albert**

USA

**Laudisio Alice**

Italy

**Sarah Amador**

UK

**Melissa K. Andrew**

Canada

**Adamantios Arampatzis**

Germany

**Hannah Arem**

USA

**Glenn Arendts**

Australia

**Joshua Armstrong**

Canada

**Claire Bamford**

UK

**John Barile**

USA

**Sarah Barnes**

UK

**Dietmar Basta**

Germany

**Alessandra Bastone**

USA

**Sarah Baum**

USA

**Iker J. Bautista**

Spain

**Marla Beauchamp**

USA

**Charlotte Beaudart**

Belgium

**Giuseppe Bellelli**

Italy

**Petra Benzinger**

Germany

**Almuth Berg**

Germany

**Emilio Berti**

Italy

**Rainer Beurskens**

Germany

**Sandipan Bhattacharjee**

USA

**Saad M. Bindawas**

Saudi Arabia

**Richard Biram**

UK

**Ingvars Birznieks**

Australia

**Hubert Blain**

France

**Alessandro Ble**

UK

**Jacques Boddaert**

France

**Thomas Boggatz**

Austria

**Kate Bolam**

Sweden

**Kenneth Boockvar**

USA

**Willem Bossers**

Netherlands

**Michael Brach**

Germany

**Marie Bradley**

USA

**Jose Bravo**

Spain

**Henry Broekhuyse**

Canada

**Thomas Buford**

USA

**Ana-Maria Buga**

Germany

**Diane Bunn**

UK

**Frances Bunn**

UK

**Elissa Burton**

Australia

**Declan Byrne**

Ireland

**Esther Calbo**

Spain

**Jan Cameron**

Australia

**Hannah Carpenter**

UK

**Maria Carrasco**

Spain

**Pilar Carrasco-Garrido**

Spain

**Margherita Cavalieri**

Austria

**Lena Cavallin**

Sweden

**Daniel Chan**

Australia

**Ding-Cheng Chan**

Taiwan

**Xueli Chen**

China

**Liang-Kung Chen**

Taiwan

**Liang-Yu Chen**

Taiwan

**Hsi-Chung Chen**

Taiwan

**Sheung-Tak Cheng**

Hong Kong

**Giorgio Chiaranda**

Italy

**Kuei-Yu Chien**

Taiwan

**Tracy Chippendale**

USA

**Carmelo Chisari**

Italy

**Mei Sian Chong**

Singapore

**Virasakdi Chongsuvivatwong**

Thailand

**Kee-Lee Chou**

China

**Chin-Liang Chu**

Taiwan

**Kevin Chui**

USA

**Veerle Claes**

Switzerland

**Andrew Clegg**

UK

**Shameka Cody**

USA

**Juan C Colado**

Spain

**Sinead Coleman**

Ireland

**Rose Collard**

Netherlands

**Simon Conroy**

UK

**Andrea Corsonello**

Italy

**Robert Cumming**

Australia

**Conal Cunningham**

Ireland

**Elizabeth Cyarto**

Australia

**Sirwan Darweesh**

Netherlands

**Piers Dawes**

UK

**Eling D. De Bruin**

Switzerland

**Jan De Lepeleire**

Belgium

**M.A.E. De Van Der Schueren**

Netherlands

**Marjolein De Vugt**

Netherlands

**Luc De Witte**

Netherlands

**Kim Delbaere**

Australia

**Panayotes Demakakos**

UK

**Wendy Den Elzen**

Netherlands

**Alexis Descatha**

France

**Mieke Deschodt**

Belgium

**Vikas Dhikav**

India

**Mario Di Napoli**

Italy

**Martin Dichter**

Germany

**Jonathan Dingwell**

USA

**Richard Dodds**

UK

**Aiden Doherty**

UK

**Lars Donath**

Switzerland

**Yi Dong**

China

**Vonetta Dotson**

USA

**Thorlene Egerton**

Australia

**Nathalie Ehrlé**

France

**Sabine Eichberg**

Germany

**Robert Eikelboom**

Australia

**Maha El Gaafary**

Egypt

**Nieke Elbers**

Australia

**Marie Elf**

Sweden

**Elisa Fabbri**

USA

**Kevin Farmer**

USA

**Véronique Faucounau**

France

**Kathleen Finlayson**

Australia

**Michael Firbank**

UK

**Leon Flicker**

Australia

**Andrea Foebel**

Sweden

**Christos Frantzidis**

Greece

**Sarah Fraser**

Canada

**Ellen Freiberger**

Germany

**Judith Fuchs**

Germany

**Joao Furtado**

Brazil

**Dominique Gagnon**

Finland

**Elizabeth Galik**

USA

**Jen-Tzer Gau**

USA

**Keith Gennuso**

USA

**Dan Gheonea**

Romania

**Clarissa Giebel**

UK

**Liz Gill**

Australia

**Muriel Gillick**

USA

**Erik Giltay**

Netherlands

**Barbara Given**

USA

**John Gladman**

UK

**Lyn Goldberg**

Australia

**Ana Gomes**

Brazil

**Fernando Gomez**

Colombia

**Adam L Gordon**

UK

**Anne Grace**

Ireland

**Elmar Graessel**

Germany

**Johannes Gräske**

Germany

**Danan Gu**

USA

**Fabio Guerini**

Italy

**Klaus Hager**

Germany

**André Hajek**

Germany

**Emily Hajjar**

USA

**Bridget Hamilton**

Australia

**Bart Hattink**

Netherlands

**Victoria Haunton**

UK

**Helen Hawley-Hague**

UK

**Allison Heid**

USA

**Stephan Heinzel**

Germany

**A. Katharina Helbig**

Germany

**Jorunn Helbostad**

Norway

**Tim Henwood**

Australia

**Hans Jürgen Heppner**

Germany

**Mira Hidajat**

USA

**Sonia Hines**

Australia

**Timo Hinrichs**

Switzerland

**Rainbow Ho**

Hong Kong

**Kara Hollowaya**

Australia

**Eva Holmgren**

Sweden

**Akira Homma**

Japan

**Emiel Hoogendijk**

Netherlands

**Lee Hooper**

UK

**Frances Horgan**

Ireland

**Fei-Yuan Hsiao**

Taiwan

**Hua-Tsen Hsiao**

Taiwan

**Amy Hsu**

USA

**Wen Hu**

China

**Guowei Huang**

China

**William Hung**

USA

**Ayumi Igarashi**

Japan

**Marco Inzitari**

Spain

**Shin-Ichi Izumi**

Japan

**Stephen Jacobs**

New Zealand

**Sabeena Jalal**

Pakistan

**Katrin Jekel**

Germany

**Jukka Jolkkonen**

Finland

**Carys Jones**

UK

**Maria Justine**

Malaysia

**Hitoshi Kagaya**

Japan

**Kei Kamide**

Japan

**Marshall Kapp**

USA

**Papatya Karakurt**

Turkey

**Christina Karlsson**

Sweden

**Eva Karlsson**

Sweden

**Amol Karmarkar**

USA

**Mark Kelson**

UK

**Gertrudis I.J.M. Kempen**

Netherlands

**Justin Keogh**

Australia

**Hunkyung Kim**

Japan

**Mijin Kim**

South Korea

**Emma Kirby**

Australia

**Renata Kirkwood**

Canada

**Naruki Kitano**

Japan

**Sofia Kjellström**

Sweden

**Byung-Joon Ko**

South Korea

**Dylan Kobsar**

Canada

**Taro Kojima**

Japan

**Stefan Kreisel**

Germany

**Reto W. Kressig**

Switzerland

**Edeltraut Kröger**

Netherlands

**Alexander Kulminski**

USA

**Ethan Kuperman**

USA

**Susan Kurrle**

Australia

**Crystal Kwan**

Canada

**Ben Lacey**

Australia

**Deirdre Lane**

UK

**Pierre Olivier Lang**

Switzerland

**Eva Langeland**

Norway

**Natasha Lannin**

Australia

**Andrew Larner**

UK

**Bobo Hi Po Lau**

Hong Kong

**Fulvio Lauretani**

Italy

**Kate Laver**

Australia

**Nanette Lavoie-Vaughan**

USA

**Siobhan Leahy**

Ireland

**Wei-Ju Lee**

Taiwan

**Xiaoyan Leng**

USA

**Angela Yee Man Leung**

China

**Paul A Lewandowski**

Australia

**Gill Lewin**

Australia

**Junxin Li**

USA

**Shanshan Li**

USA

**Lydia Li**

USA

**Xiaoli Li**

China

**Chih-Kuang Liang**

Taiwan

**Valentina Lichtner**

UK

**Nosraty Lily**

Finland

**Grace Lin**

USA

**Chiung-Ju Liu**

USA

**Hui Liu**

USA

**Laura Lorenzo Lopez**

Spain

**Stephen Lord**

Australia

**Nicole Lowres**

Australia

**Kristin Lowry**

USA

**Nan Lu**

China

**Julie Luker**

Australia

**Huabin Luo**

USA

**Ye Luo**

USA

**Hon Ming Ma**

Hong Kong

**Shylie Mackintosh**

Australia

**Janet Macneil-Vroomen**

Netherlands

**Keisuke Maeda**

Japan

**Neil Mahant**

Australia

**Rachid Mahmoudi**

France

**Andrea Maier**

Netherlands

**Jenson Cs Mak**

Australia

**Davide Malatesta**

Switzerland

**Vinaya Manchaiah**

USA

**Stéphane Mandigout**

France

**Zachary Marcum**

USA

**Alessandra Marengoni**

Italy

**James Markworth**

New Zealand

**Kara Martin**

Australia

**Manuel Martin-Carrasco**

Spain

**Yolanda Martinez**

UK

**Ferran Masanes**

Spain

**Ana Maseda**

Spain

**Daniel Matlock**

USA

**Katherine Mckenzie**

Canada

**Feng Me**

China

**Karen Devereaux Melillo**

USA

**René J.F. Melis**

Netherlands

**Qingyue Meng**

China

**Gabriele Meyer**

Germany

**Jean-Pierre Michel**

Switzerland

**Lisa Micklesfield**

South Africa

**José C. Millán-Calenti**

Spain

**Arnold Mitnitski**

Canada

**Anne-Gabrielle Mittaz Hager**

Switzerland

**Julia Morphet**

Australia

**Bente Morseth**

Norway

**Pundarik Mukhopadhaya**

Australia

**Naoki Nakaya**

Japan

**Bjørn Erik Neerland**

Norway

**Gereon Nelles**

Germany

**Alicia Nelson**

USA

**Claire Nicholl**

UK

**Francesco Nifosì**

Italy

**Anita Nilsson**

Sweden

**Yuping Ning**

China

**Prasad Nishtala**

New Zealand

**Peter Nordstrom**

Sweden

**Sarah Northcott**

UK

**Louise Nygård**

Sweden

**Izi Obokhare**

USA

**Elizabeth O'Brien**

USA

**Rónán O'Caoimh**

Ireland

**Matthew O'Connell**

Ireland

**Suguru Okubo**

Japan

**Yoshiro Okubo**

Japan

**Helian Oliveira**

Brazil

**Desmond O'Neill**

Ireland

**Francesc Orfila**

Spain

**Maria O'Sullivan**

Ireland

**Tom Overend**

Canada

**Hiroshi Oyama**

Japan

**Cemal Ozemek**

USA

**Ruth Palan Lopez**

USA

**Diana Parra**

Brazil

**H. Roeline W. Pasman**

Netherlands

**Harnish Patel**

UK

**Sophie Pautex**

Switzerland

**Ludmila Pawlikowska**

USA

**Sheila Peace**

UK

**Carlo Pedrolli**

Italy

**Jose Peeters**

Netherlands

**Monica Perracini**

Brazil

**Carol Persad**

USA

**Thien Kieu Thi Phung**

Denmark

**M. Cristina Polidori**

Germany

**Maria Lucia T. Polonio**

Brazil

**Peter Poortvliet**

Australia

**Aurel Popa-Wagner**

Germany

**Bogdan Popescu**

Romania

**Mike Price**

UK

**Juha Puustinen**

Finland

**Kylie Radford**

Australia

**Elham Rahme**

Canada

**Taina Rantanen**

Finland

**Marilyn Rantz**

USA

**Husna Razee**

Australia

**Gwyneth Rees**

Australia

**Kieran Reid**

UK

**Renan Resende**

Brazil

**Vincent Rialle**

France

**Anne-Sophie Rigaud**

France

**Stuart Ritchie**

UK

**Jongjit Rittirong**

Thailand

**Debora Rizzuto**

Sweden

**Helen Roberts**

UK

**Robert Robertson**

USA

**Lisa Robinson**

UK

**Angel Rodriguez-Laso**

Spain

**Gabriele Roehrig-Herzog**

Germany

**Roman Romero-Ortuno**

Ireland

**Isabelle Rouleau**

Canada

**Qingwei Ruan**

China

**Pierre Rumeau**

France

**Adrian Saftoiu**

Romania

**Mahshid Saghazadeh**

Japan

**Catherine Said**

Australia

**Emi Saliasi**

Netherlands

**Magnus Sandberg**

Sweden

**Lianne Sanders**

Netherlands

**Anna Sangil**

Spain

**Antonio San-Jose**

Spain

**Isabel Santana**

Portugal

**Jessica Sautter**

USA

**Laura Schaap**

Netherlands

**Daniel Schoene**

Germany

**Peter William Schofield**

Australia

**Pat Schofield**

UK

**Janice Schwartz**

USA

**Michael Schwenk**

USA

**David Scott**

Australia

**Rhonda Scudds**

Canada

**David Seamark**

UK

**Rebecca Seguin**

USA

**Andreas Seim**

Norway

**Melissa Selb**

Switzerland

**Jie Shen**

China

**Tetyana Shippee**

USA

**Sietske Sikkes**

Netherlands

**Rhian Simpson**

UK

**Mahmoud Sirdah**

Palestine

**Tina Skinner**

Australia

**Francesc Solsona**

Spain

**Yuki Soma**

Japan

**Liv Wergeland Sørbye**

Norway

**Anne Spinewine**

Belgium

**Karin Srulijes**

Germany

**Paul Stolee**

Canada

**Nimalie Stone**

USA

**Brendon Stubbs**

UK

**Pär-Daniel Sundvall**

Sweden

**Huei-Chuan Sung**

Taiwan

**Katherine Sward**

USA

**Sarah Szanton**

USA

**Hideto Takahashi**

Japan

**Balamurugan Tangiisuran**

Malaysia

**Hakan Tanriverdi**

Turkey

**Kristin Taraldsen**

Norway

**Nicholas Taylor**

Australia

**Shinji Teramoto**

Japan

**Philippe Terrier**

Switzerland

**Olga Theou**

Canada

**Katja Thomsen**

Denmark

**Petra Thürmann**

Germany

**Jochen René Thyrian**

Germany

**Anne Tiedermann**

Australia

**Suzanne Timmons**

Ireland

**Kenji Toba**

Japan

**Oly Todd**

UK

**Eline Tommelein**

Belgium

**Jose Ma Tormos Muñoz**

Spain

**Gail Towsley**

USA

**Christophe Trivalle**

France

**Taishi Tsuji**

Finland

**Mahmut Uluganyan**

Turkey

**Trinidad Valenzuela**

Australia

**Martin Vališ**

Czech Republic

**Laura Van Buul**

Netherlands

**Joost Van Hoof**

Netherlands

**Ruth Ma Van Nispen**

Netherlands

**Marielle Van Ooijen**

Netherlands

**Paula Van Wyk**

Canada

**Gianluigi Vendemiale**

Italy

**Hilde Verbeek**

Netherlands

**Beatrix Vereijken**

Norway

**Aleksandar Videnovic**

USA

**Renuka Visvanathan**

Australia

**Denese Ashbaugh Vlosky**

USA

**Hidetaka Wakabayashi**

Japan

**Jingjy Wang**

Taiwan

**Rachel Ward**

USA

**Maiko Watanabe**

Japan

**Elaine Wethington**

USA

**Rainer Wieching**

Germany

**Rainer Wirth**

Germany

**Jacek M. Witkowski**

Poland

**Karin Wolf-Ostermann**

Germany

**Bettina Wollesen**

Germany

**Rawipun Worasathit**

Thailand

**Ya-Huei Wu**

France

**Fei Wu**

USA

**Lily Xiao**

Australia

**Kuangnan Xiong**

USA

**Jianjiang Xu**

China

**Ling Xu**

USA

**Jiuping Xu**

China

**Qian Li Xue**

USA

**S Yabuki**

Japan

**Noriko Yamamoto-Mitani**

Japan

**Fang Yang**

China

**Seiji Yasumura**

Japan

**Dannii Yeung**

Hong Kong

**Zhaoxue Yin**

China

**Yoshiharu Yokokawa**

Japan

**Ji Yoo**

USA

**Jieun Yoon**

Japan

**Ju Young Yoon**

USA

**Hiroto Yoshida**

Japan

**Tatsuki Yoshimatsu**

Japan

**Jin You**

China

**Jinyoung Youn**

South Korea

**Ying Zhou**

Hong Kong

**Carolyn Zhu**

USA

**Gilbert Zulian**

Switzerland

**Franziska Zuniga**

Switzerland

**Xi-Nian Zuo**

China

**Sandra Zwakhalen**

Netherlands

